# Editorial: Interstitial Lung Disease in Primary Immunodeficiencies

**DOI:** 10.3389/fimmu.2021.699126

**Published:** 2021-07-08

**Authors:** Børre Fevang, Klaus Warnatz, John R. Hurst

**Affiliations:** ^1^ Section of Clinical Immunology and Infectious Diseases, Oslo University Hospital, Oslo, Norway; ^2^ Research Institute of Internal Medicine, Oslo University Hospital, Oslo, Norway; ^3^ Centre for Rare Diseases, Oslo University Hospital, Oslo, Norway; ^4^ Department of Rheumatology and Clinical Immunology, Medical Center- University of Freiburg, Faculty of Medicine, University of Freiburg, Freiburg, Germany; ^5^ Center for Chronic Immunodeficiency, Medical Center- University of Freiburg, Faculty of Medicine, University of Freiburg, Freiburg, Germany; ^6^ UCL Respiratory, University College London, London, United Kingdom

**Keywords:** interstitial lung disease (ILD), primary immunodeficencies (PID), common variable immune deficiency (CVID), granulomatous lymphocytic interstitial lung disease (GLILD), APECED, Autoimmune polyendocrinopathy candidiasis ectodermal dystrophy

Primary immunodeficiencies (PID) are a heterogeneous group of disorders characterized not only by increased risk of infections but also by immune dysregulation affecting a number of organs, including the lungs (1). Interstitial lung disease (ILD) in PID can therefore be considered as the pulmonary manifestation of a systemic immune dysregulation, and can be a serious threat to the health of afflicted patients (2, 3). The condition has been called granulomatous-lymphocytic interstitial lung disease (GLILD) although this term is unsatisfactory. This collection includes a broad range of articles addressing clinical, immunological and radiological features of ILD in PID.

Overall, the articles underscore the need for standardization of clinical practice and research. This is clearly shown by Van De Ven et al. presenting the findings of an international survey among pulmonologists and immunologists characterizing clinical practice and main challenges faced in care and research on GLILD. Out of 161 respondents from 47 countries only 19% had access to a standardized protocol for diagnosis and treatment. Overall, there was a wide variety in the interventions taken and the authors strongly argue for more standardized clinical studies on GLILD. Interestingly, while 71% of respondents would not routinely undertake biopsy for the diagnosis of GLILD, 46 out of 103 respondents stated that alternative diagnoses had been found on biopsies (not necessarily taken on routine), including lymphoma.

The issue of histopathological diagnosis is further explored in the review article by Dhalla et al. They argue for the need of standardization of histopathological findings to bring the understanding of the basic pathophysiology forward. There is currently considerable variation in histopathological findings between studies and we do not know if this represents biopsy-related factors or that ILD in CVID represents a spectrum of diseases, separate diseases or a shared endpoint for several diseases. An alternative strategy to understand the underlying pathophysiology can be to study bronchoalveolar lavage fluid (BAL-F). In their original article, Friedman et al., analyze findings of BAL-F from patients with common variable immunodeficiency disorders (CVID), sarcoidosis and healthy controls. They find a mixed expansion of lymphocytes in BAL-F from CVID-patients dominated by Th1-cells and CD21low B-cell while levels of regulatory T cells were low. There were also low levels of Th17-cells even if IL-17 was upregulated together with the B-cell activating factor APRIL. Mechanisms of B-cell activation, maturation and survival in the lung of affected patients are further discussed in the review article by Matson et al. The B-cell activation factor (BAFF) signaling through BAFF-R, TACI and BCMA has been shown to be associated with both presence and recurrence of ILD in CVID. The authors recommend further studies on the IFN-g/STAT1/BAFF axis.

While most articles in this topic focus on CVID, Ferré and Lionakis in their review article highlights ILD as a relevant complication of the immunodysregulatory disease Autoimmune polyendocrinopathy-candidiasis-ectodermal dystrophy (APECED). APECED can be caused by various mostly biallelic mutations in the *AIRE*-gene. Clinically and radiologically, ILD in APECED shares features with other forms of ILD, and interestingly pulmonary biopsies show a pattern of T- and B-cell infiltrates. APECED patients produce a variety of autoantibodies, including anti-BPIFB1 and anti-KCNRG that are associated with pulmonary disease. While treatment with mycophenolate and rituximab have clear clinical and radiologic effects, levels of the two autoantibodies are not affected, suggesting B-cells contribute to ILD through varied pathways, including priming of T-cells.

The issue of ILD is often raised through radiological examination and several articles look into this. Meerburg et al. examined CT scans from 138 GLILD-patients included in the STILPAD-study comparing the Baumann and Hartmann scoring methods. Both methods systematically score radiologic features of GLILD and detected the presence of features of GLILD in >95% of patients with high reproducibility, especially for the Hartmann method. The Hartmann method evaluates abnormalities in more detail than the Baumann method but is too laborious (time needed per CT scan, 30 *vs* 15 minutes, respectively) for daily clinical practice. Fraz et al., present a systematic evaluation of findings of CT and PET/CT in a cohort of 32 CVID patients with radiologic features of GLILD and relate them to clinically progressive and stable disease. Patients with progressive disease had significantly higher overall score of pathologic features on CT and higher SUV uptake on PET/CT compared to patients with clinically stable disease. Treatment with rituximab was associated with significant improvement in pathologic features while the effect on lung function measured by forced vital capacity and CO diffusion were variable.

Using data from 7 Italian PID centers Cinetto et al. present radiological, clinical and immunological findings in a cohort of 75 CVID patients with radiologic features of ILD and compare them to 125 CVID controls. The patients with radiologic ILD-findings were further divided into patients with GLILD based on histology of lung or other tissue, or undetermined (u)ILD based on a clinical-radiological diagnosis without biopsy. Patients with GLILD and to a lesser extent uILD were characterized by splenomegaly, autoimmune cytopenia, low DLCO and high frequency of CD21low B-cells. Pooling these features together the authors made a predictive model for GLILD with a ROC curve of 0.98, possibly limiting the need for diagnostic biopsies for GLILD. Lopes et al. present clinical, immunologic and radiologic data on 46 patients with biopsy-proven ILD. They find a rate of granulomas above 50% in pulmonary tissue but also high frequency of lymphoid interstitial pneumonia. Nine patients died during the observation period with a median age of death of 49 years underscoring the serious nature of this complication in CVID.

There is therefore a clear need for better treatment of ILD in PID, and in their systematic review article, Lamers et al., aim to summarize and synthesize literature on efficacy of treatments for GLILD. They find 41 papers describing case series or uncontrolled studies reporting on 255 patients. The heterogeneity characterizing publications on GLILD makes comparing studies difficult but there was a trend towards more relapses in patients treated with glucocorticoids only. van Stigt et al. approach treatment of GLILD through looking at treatment of granulomatous disease of CVID in general in their review article. Extra-pulmonary disease has been reported in lymph nodes, spleen, gastrointestinal tract, bone marrow, liver and skin among others. Reports of 95 CVID-patients treated for granulomatous disease were identified in literature (45 patients with extra-pulmonary disease only, 51 patients with pulmonary granulomatous disease) receiving a total of 117 different treatment courses. While steroid monotherapy is used for all granulomatous disease, it is reported more frequently for extrapulmonary disease (21/53 *vs* 15/64 courses, extrapulmonary and pulmonary disease, respectively) and with remission in 85.7% of cases. Anti-TNF therapy was also more frequently reported in extrapulmonary disease, while rituximab and azathioprine were administered almost solely in pulmonary disease.

This Research Topic will hopefully inspire centers around the world to collaboratively tackle this field. An important first milestone will be to agree on which criteria to base the diagnosis of ILD in PIDs (**Figure 1**).

**Figure 1:**
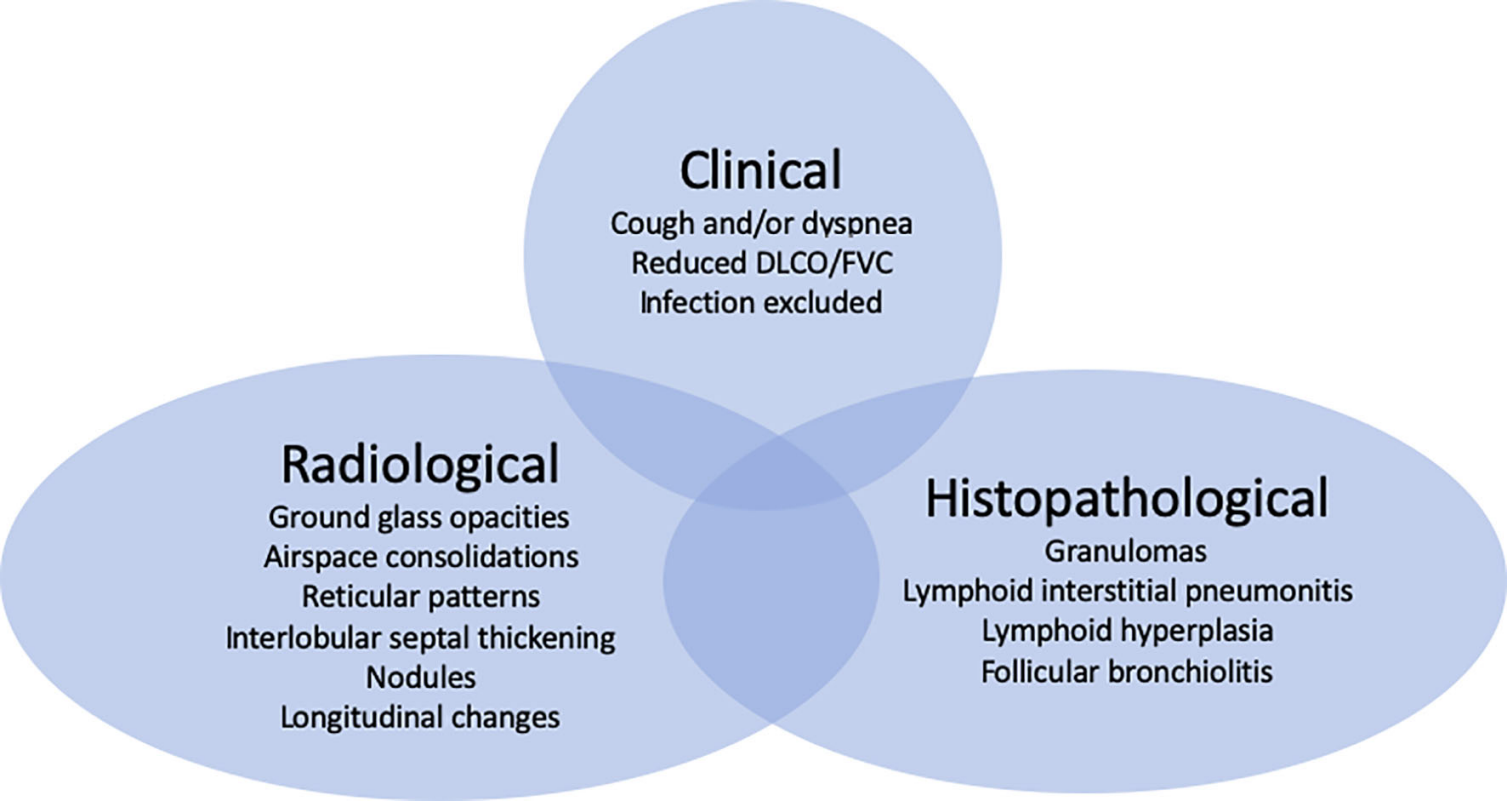
(GL) ILD in CVID is characterized by clinical, radiological and histopathological features with patients presenting all or some of these manifestations. Which criteria to include for the diagnosis demands an imminent debate (4).

## Author Contributions

BF, KW, and JH all contributed to the planning and writing of this manuscript. BF wrote the first draft, which was then revised by KW and JH. All authors contributed to the article and approved the submitted version.

## Conflict of Interest

The authors declare that the research was conducted in the absence of any commercial or financial relationships that could be construed as a potential conflict of interest.
